# Thin Reinforced Ion-Exchange Membranes Containing Fluorine Moiety for All-Vanadium Redox Flow Battery

**DOI:** 10.3390/membranes11110867

**Published:** 2021-11-11

**Authors:** Ha-Neul Moon, Hyeon-Bee Song, Moon-Sung Kang

**Affiliations:** Department of Green Chemical Engineering, Sangmyung University, Cheonan 31066, Korea; gksmf1385@gmail.com (H.-N.M.); gusql1231@gmail.com (H.-B.S.)

**Keywords:** pore-filled ion-exchange membranes, all-vanadium redox flow battery, hydrocarbon-based ionomer, fluorine moiety, porous polyethylene substrate, oxidation stability

## Abstract

In this work, we developed pore-filled ion-exchange membranes (PFIEMs) fabricated for the application to an all-vanadium redox flow battery (VRFB) by filling a hydrocarbon-based ionomer containing a fluorine moiety into the pores of a porous polyethylene (PE) substrate having excellent physical and chemical stabilities. The prepared PFIEMs were shown to possess superior tensile strength (i.e., 136.6 MPa for anion-exchange membrane; 129.9 MPa for cation-exchange membrane) and lower electrical resistance compared with commercial membranes by employing a thin porous PE substrate as a reinforcing material. In addition, by introducing a fluorine moiety into the filling ionomer along with the use of the porous PE substrate, the oxidation stability of the PFIEMs could be greatly improved, and the permeability of vanadium ions could also be significantly reduced. As a result of the evaluation of the charge–discharge performance in the VRFB, it was revealed that the higher the fluorine content in the PFIEMs was, the higher the current efficiency was. Moreover, the voltage efficiency of the PFIEMs was shown to be higher than those of the commercial membranes due to the lower electrical resistance. Consequently, both of the pore-filled anion- and cation-exchange membranes showed superior charge–discharge performances in the VRFB compared with those of hydrocarbon-based commercial membranes.

## 1. Introduction

As energy demand is rapidly increasing around the world and environmental pollution caused by the use of fossil fuels is emerging, renewable energy is attracting attention as the energy source of the future. However, renewable energy has a disadvantage in that the output fluctuates greatly depending on the climatic environment, and to compensate for this, an energy storage system (ESS) with high capacity is required [1,2]. ESS is a key component of a smart grid, and various types of secondary batteries that can be used for a long time and have high energy efficiency during operation are mainly used for large-capacity energy storage [1,2,3]. That is, lithium-ion batteries, lead-acid batteries, NaS batteries, and redox flow batteries (RFBs) are known to be efficient secondary batteries for ESS applications. Among them, RFBs possess higher availability and energy efficiency and lower capital cost requirements than other competing technologies. In addition, they are believed to have several advantages, such as low toxicity and long lifespan. In particular, the RFBs operate at room temperature, and the power and capacity of the battery can be designed independently of each other [3].

**Figure 1:**
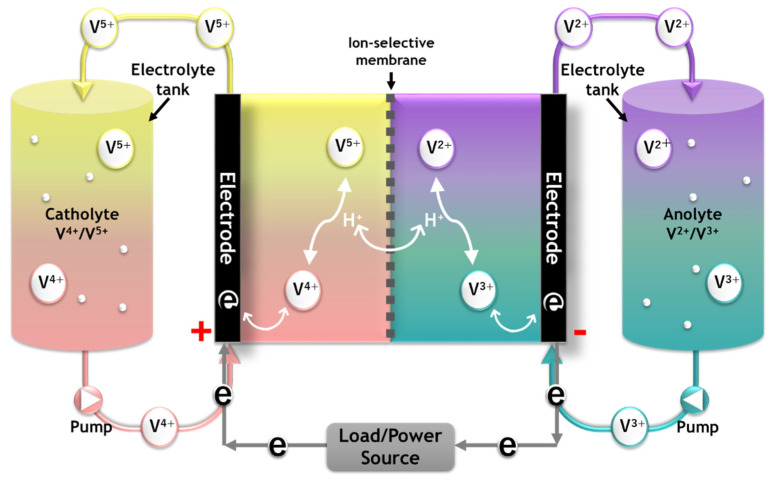
Schematic diagram of a VRFB system showing the working principle.

The RFB is a battery system in which an active electrode material dissolved in an electrolyte solution is oxidized and reduced to charge and discharge. In more detail, after dissolving the cathode and anode active materials in the electrolyte, they are respectively stored in an external tank and circulated through the stack using a pump when necessary, and electric energy is charged and discharged. Undesirable mixing of the electrolyte components can be prevented by independent storage of cathode and anode active materials, and a high level of stability can be obtained by using an aqueous electrolyte. As an active material for the RFB, redox couples with various potentials, such as iron/chromium, iron/titanium, all vanadium, vanadium/bromine, polysulfide bromine, zinc/bromine, and zinc/cerium, can be selected [4,5,6]. In the case of having different redox ion species at the cathode and the anode, such as an iron/chromium system, significant cross contamination of electrolytes can occur due to the concentration gradient of each species in the two sides of the membrane [7]. Since the crossover of these active materials causes self-discharge and limits the RFB performance, a technique using the same species as redox couples for both the anode and cathode has been proposed [7,8]. A typical example is an all-vanadium flow battery (VRFB) using vanadium species as both the anode and the cathode redox materials. The VRFB has several advantages, such as excellent energy efficiency, long lifespan, and high cost-effectiveness. Figure 1 shows the structure and charge–discharge principle of a typical VRFB system.

Meanwhile, the membrane is one of the most important components that determine the charge–discharge performance and durability of all kinds of RFB systems [9]. Although it depends on the type of redox couples, RFBs mainly employ ion-exchange membranes (IEMs) that prevent the mixing of electrolytes between the anode and cathode compartments and act as an ion conductor [2,5]. The IEMs used in the RFB system require low electrical resistance, high selective permeability for specific ions, low diffusion coefficient for solvents, and excellent chemical and mechanical stabilities [10]. Considering the characteristics of the VRFB system operated under strongly acidic conditions, the IEMs should also have high acid and oxidation resistance and excellent selective permeability to hydrogen ions compared with vanadium cations [10,11]. From this point of view, Nafion, a perfluorinated cation-exchange membrane (CEM), has been widely utilized as a separation membrane in VRFB systems, but it is suffering from some drawbacks, such as high membrane cost and significant vanadium crossover. As an alternative, therefore, the use of anion-exchange membranes (AEMs) has recently attracted attention, and hydrocarbon-based IEMs are being actively developed to lower the expensive membrane cost [12,13]. However, in the case of hydrocarbon-based IEMs, despite their excellent electrochemical characteristics, they are difficult to apply to practical systems due to their poor chemical stability, so research on this is urgently needed [2,10,14,15]. In addition, a partially fluorinated IEM can be considered to improve the chemical stability and reduce the manufacturing cost of the IEMs [16,17,18,19,20].

**Figure 2:**
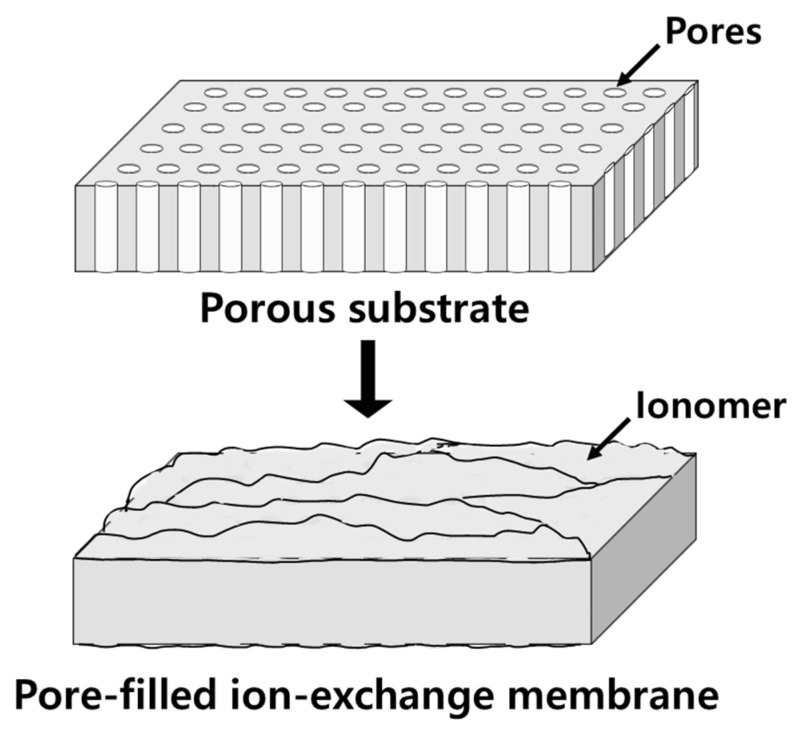
Schematic drawing of PFIEM fabrication.

The traditional manufacturing process (i.e., “a paste method”) of commercial IEMs is known to be complicated and expensive. In this method, typically, the IEMs are fabricated by impregnating a paste mixed with monomers and rubber into a reinforcing fabric net, radical polymerization, and then introducing ion-exchange groups through a post-treatment, such as quaternization (for AEMs) or sulfonation (for CEMs) [21]. Meanwhile, a pore-filled ion-exchange membrane (PFIEM), in which an ionomer is filled into pores of a thin porous polymer film, is shown to possess low mass transport resistance and strong mechanical strength, so it is being considered for application to various energy conversion technologies and water treatment processes [22,23,24]. The PFIEM, which is intermediate between a homogeneous membrane and a heterogeneous membrane, exhibits excellent electrochemical properties while lowering the manufacturing cost due to the use of inexpensive reinforcing material and a reduction in the amount of raw materials used. Figure 2 illustrates the fabrication principle of the PFIEM.

**Figure 3:**
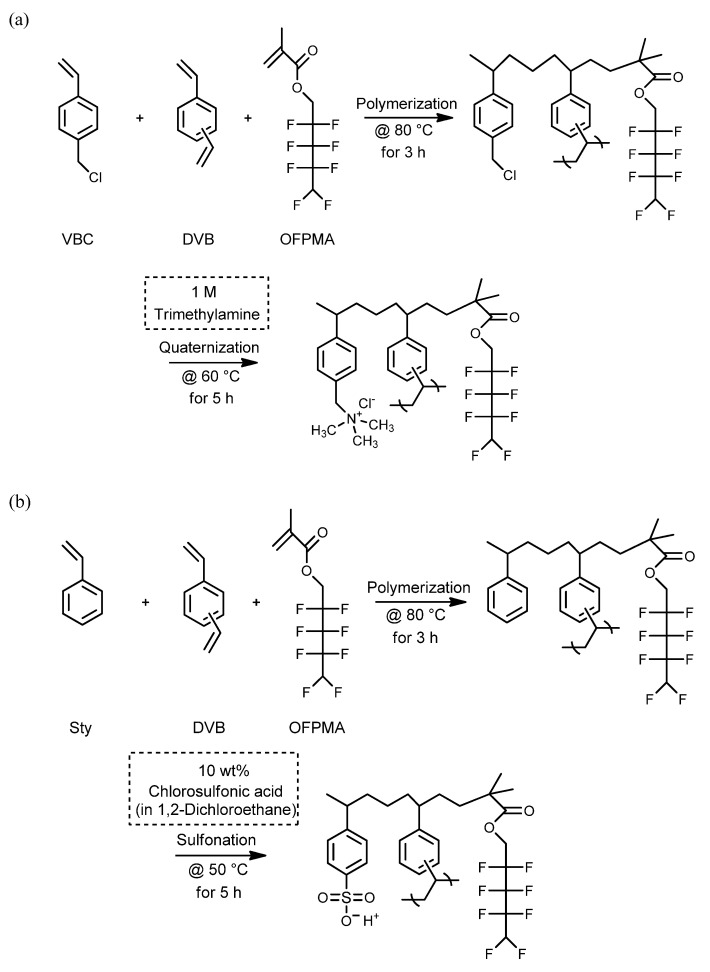
Reaction scheme of (**a**) quaternized anion-exchange polymer and (**b**) sulfonated cation-exchange polymer.

In this study, novel IEMs optimized for VRFB application were developed by combining an ionomer with a porous polyethylene (PE) substrate as a reinforcing material. It was expected that the PFIEMs could possess low electrical resistance, excellent chemical and physical stabilities, and low production cost by employing a simple pore-filling method. Figure 3 shows the synthesis process of the anion- and cation-exchange polymers prepared. For the membrane fabrication, 4-vinylbenzyl chloride (VBC) or styrene (Sty), the main monomer; benzoyl peroxide (BPO), a thermal initiator; and divinylbenzene (DVB), a cross-linking agent, were filled in the pores of a porous PE substrate, and a base membrane was then prepared through in situ radical polymerization. The prepared base membrane was followed by quaternization or sulfonation post-treatment to produce a pore-filled anion-exchange membrane (PFAEM) and a pore-filled cation-exchange membrane (PFCEM), respectively. In addition, 1H,1H,5H-octafluoropentyl methacrylate (OFPMA) monomer was mixed with the monomer solution to prepare PFIEMs with a fluorine moiety. The OFPMA employed is a chemically robust fluorine monomer widely used in surface coatings to prevent oxidation, and is characterized by low *T_g_* and low surface energy [25,26,27,28]. The introduction of fluorine groups was expected to effectively improve the chemical stability of the PFIEMs under strongly acidic and oxidative conditions of VRFB. In this work, in particular, the effect of the content of a fluorine moiety on membrane properties and VRFB performance was systematically investigated, and the characteristics of PFAEMs and PFCEMs were also compared.

## 2. Materials and Methods

### 2.1. Materials and Membrane Preparation

A porous PE film (Hipore, *t* = 25 μm) was supplied by Asahi Kasei (Japan) and used as a reinforcing material for preparing the PFIEMs. The specifications for the commercial porous PE substrate used in this work were found from the literature and are summarized in Table 1 [29]. As described previously, VBC and/or Sty were used as main monomers for introducing ion-exchange groups. BPO and DVB were employed as a thermal polymerization initiator and a cross-linking agent, respectively. For the quaternization and sulfonation of base membranes, trimethylamine (TMA) and chlorosulfonic acid (CSA) were used, respectively, and 1,2-dichloroethane was employed as a solvent. As a monomer containing a fluorine moiety, OFPMA was purchased from TCI (Japan) and employed. All reagents except OFPMA were purchased from Sigma-Aldrich (USA) and were used as received without any purification. In addition, AMX and CMX (Astom Corp., Japan) were chosen as the commercial hydrocarbon-based AEM and CEM, respectively, for membrane property comparison.

For the membrane fabrication, a porous PE film was first impregnated with a monomer mixture. At this time, the monomer mixture was prepared with VBC and/or Sty and OFPMA at molar ratios of 1:0, 2:1, 3:1, and 4:1, respectively. In addition, 20 wt% of DVB as a cross-linking agent and 2 wt% of BPO as an initiator were added and fully mixed using a magnetic stirrer. The detailed composition of the monomer mixture is summarized in Table 2. After that, the monomer-filled substrate film was heated at 80 °C for 3 h in an oven for the thermal radical polymerization, producing a base membrane. For the fabrication of the PFAEM, the base membrane was then immersed in 1.0 M TMA aqueous solution, followed by a quaternization reaction at 60 °C for 5 h. Similarly, the PFCEM was prepared by reacting the base membrane in 10 wt% CSA (in 1,2-dichloroethane) solution at 50 °C for 5 h. The prepared PFIEMs were washed with distilled water and ethanol and stored in 0.5 M NaCl aqueous solution before use.

### 2.2. Membrane Characterizations

The membrane electrical resistance (MER) of IEMs is related to the internal resistance of the VRFB system and is a factor that dominantly determines the voltage efficiency (VE). To measure the MER, the membrane sample was first immersed in 0.5 M NaCl solution for at least 5 h to reach its equilibrium state. First, the blank resistance (*R_2_*) of the 0.5 M NaCl solution was determined using a lab-made clip cell connected to a potentiostat/galvanostat with electrochemical impedance spectroscopy (SP-150, Bio-Logic Science Instruments, France). The membrane sample was then inserted into the clip cell and immersed in 0.5 M NaCl solution to measure the membrane + solution resistance (*R_1_*). Finally, the membrane electrical resistance was calculated by substituting the measured *R_1_* and *R_2_* values into the following Equation (1) [30]:

(1) $$\mathrm{MER}=\left(R_{1}-R_{2}\right)\times A\;\left[\Omega \cdot {\mathrm{cm}}^{2}\right]$$

where *A* is the effective area of Pt electrodes constituting the clip cell. Meanwhile, the ion conductivity (*σ*) of the IEMs was obtained from the following Equation (2) [31]:

(2) $$\sigma =\frac{L}{\mathrm{MER}}\;\left[\frac{S}{\mathrm{cm}}\right]$$

where *L* is the thickness of the membrane sample.

To measure the water uptake (WU), a membrane sample having a size of 2 × 2 cm^2^ was immersed in distilled water to reach an equilibrium state. After removing the water present on the sample surface with filter paper, the wet weight (*W_wet_*) was measured and then dried in a dry oven at 80 °C for more than 6 h to measure the dry weight (*W_dry_*). The WU values were determined using the following Equation (3) [31]:

(3) $$WU=\frac{W_{wet}-W_{dry}}{W_{dry}}\times 100\;\left[\%\right].$$

The swelling ratio (*SR*) of the prepared membranes was determined with the following Equation (4) by measuring the volume of the dried membrane (*V_dry_*) and the volume of the wet membrane (*V_wet_*, swelled with the electrolyte solution used for VRFB tests) [31]:

(4) $$SR=\frac{V_{wet}-V_{dry}}{V_{dry}}\times 100\;\left[\%\right].$$

The ion-exchange capacity (IEC) of the prepared membranes was measured with a sample having a size of 2 × 2 cm^2^. In the case of an AEM, the sample was immersed in 0.5 M NaCl solution for more than 6 h so that the ion-exchange groups were exchanged with Cl^-^, and then washed several times with distilled water, and the wet weight was measured. Then, it was immersed in 0.25 M Na_2_SO_4_ aqueous solution for 3 h or more so that Cl^−^ ions of the ion-exchange groups were fully replaced with SO_4_^2−^ ions. The amount of Cl^−^ present in the solution was determined by Mohr’s method using a K_2_CrO_4_ indicator and 0.01 M AgNO_3_ aqueous solution as titrant. In the case of a CEM, the sample was immersed in 0.5 M HCl solution for more than 6 h to reach an equilibrium state. After washing with distilled water, the sample was immersed in 0.5 NaCl solution for 3 h or more so that Na^+^ was exchanged with H^+^ present in the sample. The amount of H^+^ existing in the solution was then determined by a traditional acid–base titration using a phenolphthalein indicator and a 0.01 M NaOH titration solution. Finally, the IEC value of the sample was calculated by substituting the measured parameters into the following Equation (5) [31]:

(5) $$\mathrm{IEC}=\frac{C\cdot V_{s}}{W_{dry}}\;\left[\frac{\mathrm{meq}.}{g_{\mathrm{dry}\;\mathrm{memb}}}\right]$$

where *C* is the normal concentration of ions measured through titration (meq./L), *V_s_* is the solution volume (L), and *W_dry_* is the dry membrane weight (g).

The transport numbers (*t_−_* for anion and *t_+_* for cation) of the IEMs were determined by measuring the membrane potential using a pair of Ag/AgCl electrodes in a two-compartment diffusion cell and calculated by the following Equations (6) and (7) [32]:

(6) $$E_{m}=\frac{RT}{F}(2t_{+}-1)\ln\frac{C_{L}}{C_{H}}$$

(7) $$t_{+}+t_{-}=1$$

where *E_m_* is the measured membrane potential, *R* is the gas constant, *T* is the absolute temperature, *F* is the Faraday constant, and *C_L_* and *C_H_* are NaCl concentrations of the compartments (1 and 5 mM, respectively).

To evaluate the oxidation stability of the prepared IEMs, a membrane sample (2 × 2 cm^2^) was immersed in an aqueous solution containing 3% H_2_O_2_ and 3 ppm Fe^2+^ (i.e., Fenton’s reagent), and then the reaction proceeded at 80 °C for 8 h. During the oxidation test, the membranes could be decomposed by free radicals (·OH and ·OOH) formed by H_2_O_2_ in the presence of Fe^2+^ ions [33]. Therefore, each sample reacted for 0 (fresh), 4, and 8 h was washed several times with distilled water and dried in a dry oven at 80 °C for more than 6 h, and then the weight was measured to confirm the weight loss of the sample.

Additionally, it was attempted to confirm the chemical stability of the IEMs in the VRFB system through a vanadium oxidation stability test. This is based on the principle that VO_2_^+^, which is a V(V) species, is reduced to VO^2+^, which is a V(IV) species, by an oxidation reaction of a film immersed in a VO_2_^+^/H_2_SO_4_ solution. For the measurement of vanadium oxidation stability, a membrane sample (2 × 2 cm^2^) was immersed in 20.0 mL of 0.1 M V_2_SO_5_ (in 5 M H_2_SO_4_) solution, and the temperature was maintained at 40 °C. The concentration of VO^2+^ ions in the solution was determined by measuring the absorbance using UV–VIS spectroscopy (UV-2600, Shimadzu).

A permeability test was also carried out to confirm the vanadium crossover through the IEMs. A membrane sample having a size of 5 × 5 cm^2^ was immersed in a 2 M H_2_SO_4_ solution for 2 h or more to reach an equilibrium state, and then inserted in a lab-made two-compartment diffusion cell. Amounts of 2 M VOSO_4_/2 M H_2_SO_4_ (feed) and 2 M MgSO_4_/2 M H_2_SO_4_ (permeate) solutions were filled in each compartment. The time-course change in the VO^2+^ ion concentration was then determined by measuring the absorbance using UV–VIS spectroscopy, and the permeability (overall dialysis coefficient, *K_A_*) of the VO^2+^ ion through the IEM was calculated using the following Equation (8) [34]:

(8) $$K_{A}=\frac{k_{V}}{1+k_{V}}\frac{V^{II}}{At}\ln\frac{c_{A0}^{I}}{c_{A0}^{I}-\frac{1+k_{V}}{k_{V}}c_{A}^{II}}\;\left[\frac{m}{s}\right]$$

where $C_{A}^{I}$ and $C_{A}^{II}$ are the molar concentration of component A (i.e., VO^2+^) in feed (I) and permeate (II) compartments, respectively; $C_{A0}^{I}$ is the initial molar concentration of component A in the feed compartment; *A* is the membrane effective area, $V^{I}$ and $V^{II}$ are the solutions volume in feed (I) and permeate (II) compartments, respectively; *k_v_* is the solution volume ratio of both compartments (= $V^{I}$/$V^{II}$); and *t* is time.

The mechanical strength of commercial and prepared IEMs was evaluated according to international standards (ASTM method D-882-79) using a universal testing machine (5567 model, Instron).

### 2.3. VRFB Performance Tests 

The evaluation of the charging–discharging performance of the VRFB was performed using a lab-made RFB unit cell. A 2.0 M V_2_(SO_4_)_3_/2.0 M H_2_SO_4_ aqueous solution was used as the cathode electrolyte, and a 2.0 M VOSO_4_/2.0 M H_2_SO_4_ aqueous solution was employed as the anode electrolyte. Carbon felt (GF20-3, Nippon Graphite) was used as the electrode, and the effective area of the electrode and membrane was 12.5 cm^2^. Using an automatic battery cycler (WBCS 3000, Wonatech), it was charged to 1.9 V at a current density of 20 mA/cm^2^ and then discharged to 0.9 V. Coulombic efficiency (CE), VE, and energy efficiency (EE) for charging–discharging performance evaluation were calculated through the following Equations (9)–(11), respectively.

(9) $$\mathrm{CE}=\frac{\mathrm{Discharge}\;\mathrm{capacity}\;\left(\mathrm{Ah}\right)}{\mathrm{Charge}\;\mathrm{capacity}\;\left(\mathrm{Ah}\right)}\times 100\;\left[\%\right]$$

(10) $$\mathrm{VE}=\frac{\mathrm{Average}\;\mathrm{discharge}\;\mathrm{voltage}\;\left(V\right)}{\mathrm{Average}\;\mathrm{charge}\;\mathrm{voltage}\;\left(V\right)}\times 100\;\left[\%\right]$$

(11) EE=CE×VE [%]

## 3. Results and Discussion

**Figure 4:**
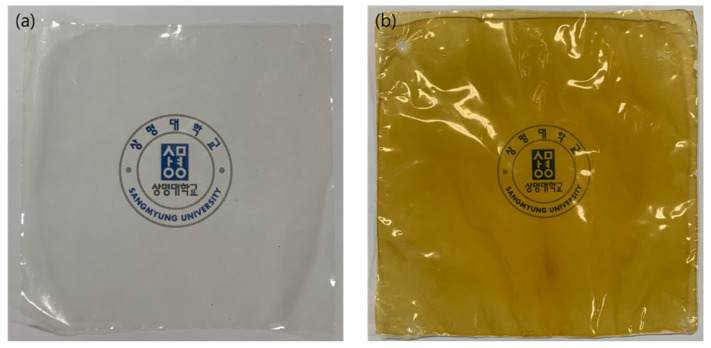
Pictures of (**a**) pore-filled anion-exchange membrane (PFAEM-1) and (**b**) pore-filled cation-exchange membrane (PFCEM-1).

Figure 4 shows photographs of the prepared PFIEMs. Unlike the opaque porous substrate film, it can be seen that the prepared PFIEMs are shown to be transparent, and from this, it can be indirectly confirmed that the pores of the substrate are completely filled with a polymer. In addition, in the case of PFCEM, the color was changed to dark yellow after the post-treatment, and therefore, it can be expected that a sulfonation reaction occurred in the pore-filled polymer.

The content of ionomer filled in the porous substrate is measured and summarized in Table 3. The content of the filled ionomer is similar to the porosity of the porous substrate shown in Table 1.

**Figure 5:**
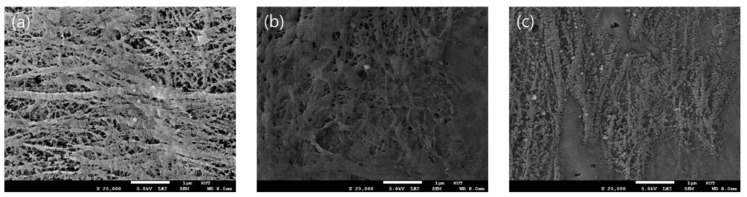
FE-SEM images (surface, ×20,000) of (**a**) porous PE substrate, (**b**) PFAEM-1, and (**c**) PFCEM-1.

In addition, field-emission scanning electron microscopy (FE-SEM, JSM-7500F, JEOL Ltd., Japan) analysis was performed to check the surface morphology of the prepared IEMs. As shown in Figure 5, it was confirmed that the pores of the porous substrate were completely filled with a polymer after the membrane preparation, and there were no open pores [35].

**Figure 6:**
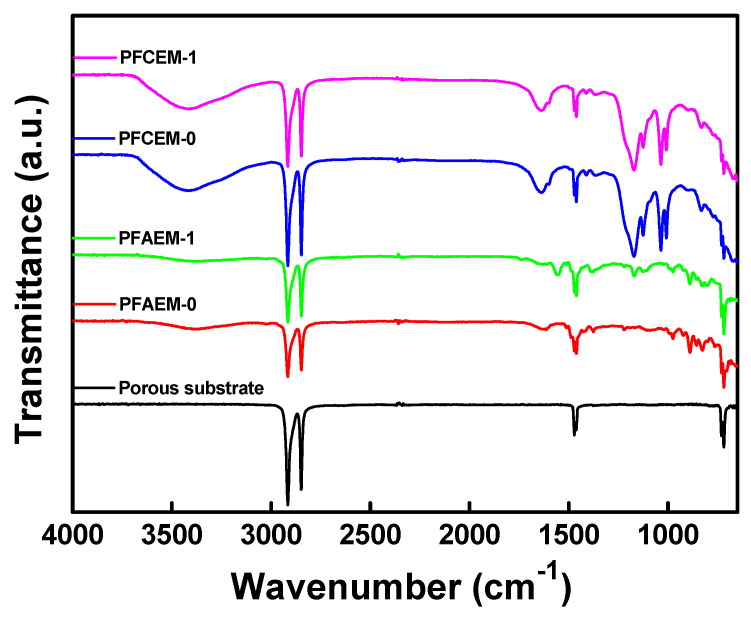
FTIR spectra of porous substrate, PFAEMs, and PFCEMs.

The FTIR spectra of the porous PE substrate and the prepared PFAEMs and PFCEMs are shown in Figure 6. In the FTIR spectra of PFAEM, the successful introduction of quaternary ammonium groups was confirmed from the absorption peaks observed at 1372, 975, 890, and 812 cm^−1^ [36,37,38,39]. Additionally, the presence of C=C bonds and aromatic rings was checked from the absorption peaks found at 1640 and 1390 cm^−1^, respectively [40,41]. Meanwhile, CF_2_ stretching vibration was observed at 1170 cm^−1^ in the spectrum of PFAEM-1 [42]. In addition, C=O and C-O-C bonds were confirmed at 1740 and 1120 cm^−1^, respectively, indicating the introduction of a fluorine moiety due to the copolymerization of OFPMA monomer [43,44]. Meanwhile, in the spectra of PFCEM, the absorption peaks assigned to sulfonic acid groups were found at 1127, 1037, 1008, and 678 cm^−1^, elucidating the successful introduction of cation-exchange groups [38]. In the case of the PFCEM, the existence of the fluorine moiety could not be checked from the FTIR spectra due to the overlapping of the absorption bands. Overall, it can be demonstrated that the preparation of the PFAEM and PFCEM was successfully performed through the monomer pore filling, in situ radical polymerization, and post-treatment reaction.

**Figure 7:**
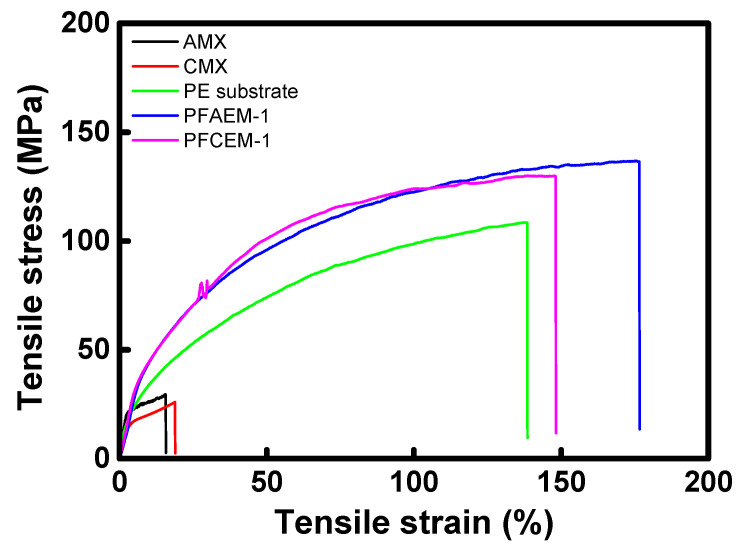
Tensile stress–stain curves of commercial IEMs, porous substrate, and PFIEMs.

The IEMs used in the RFB are required to have excellent mechanical strength to resist pressure drop owing to high flow rates. Tensile strength and elongation at break are important parameters indicating the mechanical strength of IEMs [45]. The results of tensile strength and stress measurement for the commercial membranes, the porous substrate film, and the prepared PFIEMs are summarized in Figure 7 and Table 4. It can be seen that the porous PE film used as the reinforcing material has an excellent tensile strength (125.1 MPa) and elongation at break (46.47%) despite a relatively thin film thickness compared with those of commercial IEMs. In addition, the prepared PFIEMs revealed largely improved tensile strength and toughness compared with the porous substrate film. From the results, it was confirmed that the PFIEMs fabricated in this work had superior mechanical strength despite having a thickness of about 1/6 of the commercial membranes.

Various characteristics of the commercial IEMs and the prepared PFIEMs are summarized in Table 5. In this study, the PFIEMs were prepared according to the molar ratio of VBC, Sty, and OPFMA, and then the membrane properties were systematically evaluated. As the mole ratio of VBC or Sty is increased, the portion of OPFMA is relatively decreased, and therefore, the content of the fluorine part in the membrane is reduced. The amount of ion-exchange groups introduced into VBC or Sty increased while decreasing the fluorine part, resulting in an increase in the IEC. In addition, it can be seen that the WU and *σ* of the PFIEMs increased, and the electrical resistance decreased due to the increase in the IEC. The high elongation of the prepared membrane was due to the intrinsic characteristics of the porous PE support used, which means that it could be stretched when a strong external force is continuously applied. However, it was proven that the excessive swelling of the membranes did not occur in actual use based on the SR data shown in Table 5. Meanwhile, the *σ* of the PFIEMs showed a lower value compared with the commercial membranes because the non-ion conducting area was greatly increased due to the use of an inert PE substrate like in the case of a heterogeneous IEM. However, all the PFAEMs and PFCEMs fabricated in the considered composition range showed significantly lower electrical resistance compared with the commercial IEMs, which was mainly due to the relatively thin film thickness. As a result of measuring the surface contact angle, it can be observed that the hydrophobicity of the PFIEMs containing a fluorine moiety was somewhat higher than that of the commercial membranes, which is thought to be owing to the characteristic of the PE substrate with strong hydrophobicity (i.e., contact angle of the PE substrate = ca. 99.0 degree) [46]. It can be seen that the prepared PFIEMs generally exhibited a low water content and high surface hydrophobicity compared with the commercial membranes, which was considered to be advantageous in reducing the crossover of vanadium ions and increasing the oxidation stability of the membrane.

**Figure 8:**
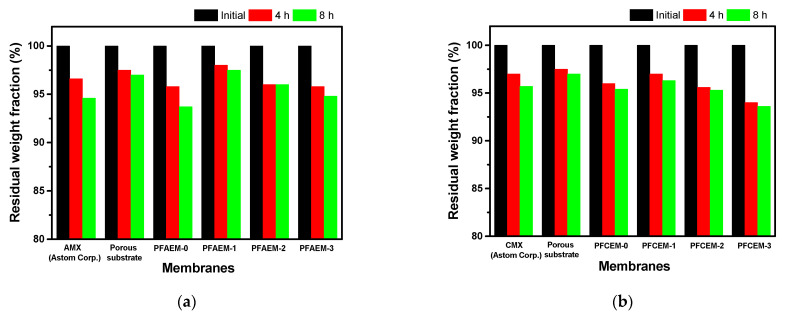
Time course changes in residual weight fraction during the Fenton oxidation test of (**a**) AEMs and (**b**) CEMs.

Figure 8 exhibits the results of measuring the weight change of the IEMs during the Fenton oxidation test. It can be seen that PFAEM-1 and PFCEM-1 containing the most fluorine moieties exhibited the best oxidation stability among the membranes tested. The data demonstrate that the oxidation stability elevated as the content of the fluorine moiety increased. Therefore, it can be confirmed that the oxidation stability of the PFIEMs was improved due to the introduction of the fluorine moiety. However, PFCEM-2 and PFCEM-3 showed lower oxidation stability compared with PFCEM-0 without a fluorine moiety. In the case of poly(styrenesulfonic acid), it is known that chain scission by the HO⸱ radical is promoted at low pH conditions [47,48]. That is, it is believed that the high content of sulfonic acid groups (i.e., high acidity) promotes the oxidative degradation of a cation-exchange polymer.

**Figure 9:**
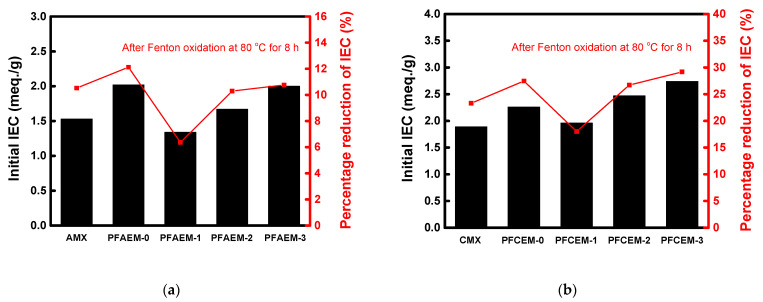
Initial IEC values and percentage reduction of IEC after Fenton oxidation at 80 °C for 8 h of (**a**) AEMs and (**b**) CEMs.

The percentage reduction of the membrane IEC after the Fenton oxidation experiment was determined, and the results are shown in Figure 9. As a result, it was found that PFAEM-1 and PFCEM-1, which had the highest fluorine content among the membrane samples tested, showed the same tendency with the change in weight and had the lowest IEC reduction rate. This result demonstrates that the fluorine moiety introduced into the membranes can also improve the oxidative stability of the ion-exchange groups.

**Figure 10:**
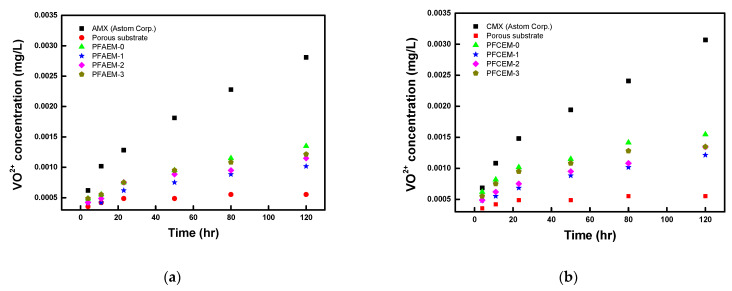
Time course changes in VO^2+^ concentration during the chemical stability test of (**a**) AEMs and (**b**) CEMs in 0.1 M V_2_SO_5_ (in 5 M H_2_SO_4_).

Meanwhile, IEMs applied to a VRFB operating under strongly acidic conditions may cause problems, such as a decrease in the degree of cross-linking and decomposition of functional groups when used for a long period of time [49]. Therefore, the chemical stability of the prepared PFIEMs by measuring the rate of membrane decomposition in a vanadium electrolyte solution was also evaluated. When immersed in a VO_2_^+^/H_2_SO_4_ solution, VO_2_^+^ is reduced to VO^2+^ due to the oxidation reaction of the membrane. Thus, there is a proportional relationship between the generation rate of VO^2+^ ions and the decomposition rate of the IEMs [50]. Figure 10 shows the results of measuring the concentration of VO^2+^ generated by the oxidation reaction of each IEM. As a result, it was confirmed that the oxidation stability of the PFIEMs in the vanadium electrolyte was significantly higher than that of the commercial membranes. This is considered to be a result of the excellent stability of the porous PE film used as the reinforcing material. In addition, as the ratio of the fluorine moieties increased, a lower VO^2+^ ion production rate was exhibited. From this, it was also confirmed that the chemical stability of the IEMs for VRFB application could be improved by introducing fluorine moieties into the membrane.

Vanadium ion permeability through a membrane is a parameter indicating the crossover characteristic of vanadium ions. The crossover of vanadium ions as the active electrode material can be regarded as a self-discharge process in a VRFB system. Vanadium ions pass through the IEM and chemically react with vanadium ions of different oxidation numbers, resulting in efficiency loss and capacity reduction [51]. Since the crossover behavior is mainly determined by the membrane characteristics, fabricating the IEMs with a reduced crossover rate is one of the important issues in the RFB technology field. An ideal IEM for VRFB should have low vanadium ion permeability to reduce self-discharge and achieve high current efficiency [52]. The vanadium ion permeability values of the commercial membranes and the prepared PFIEMs are listed in Table 5. As a result, it was confirmed that the permeability of vanadium ions increased as the ratio of VBC and Sty in the prepared PFIEMs increased. That is, the permeability of the vanadium active material is elevated because the water uptake and the free volume increase by increasing the IEC in the membranes. However, despite the thin film thickness compared with the commercial membranes, the vanadium ion permeability in the PFIEMs was shown to be relatively low due to the low water uptake (wettability) and surface hydrophilicity. As a result, the vanadium ion permeability was lowest when the ratio of fluorine moieties was highest in both the PFAEMs and PFCEMs. In addition, when comparing both types of IEMs, it can be seen that the vanadium ion permeability in the PFAEMs is relatively low compared with that of the PFCEMs. This result demonstrates that in the case of the PFAEMs, the crossover of cations (i.e., vanadium ions) can be effectively reduced by means of the Donnan exclusion.

**Figure 11:**
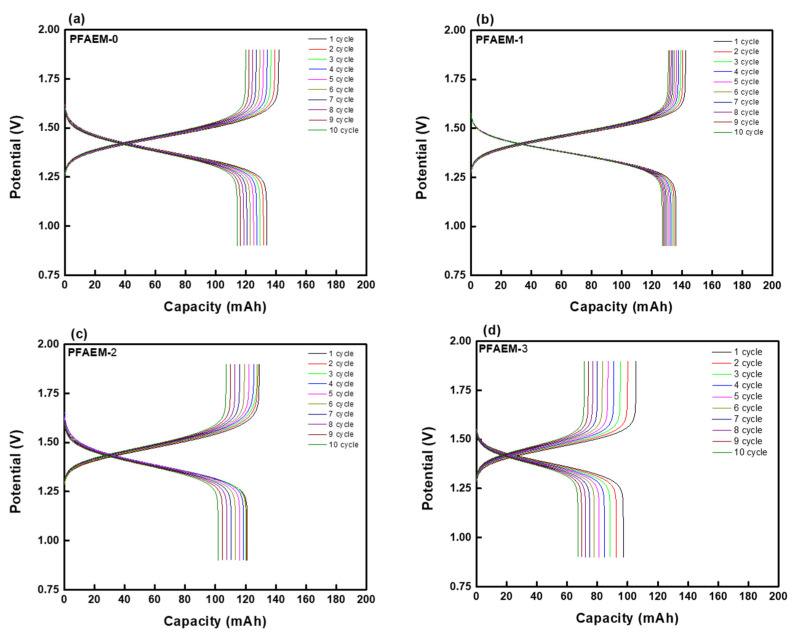

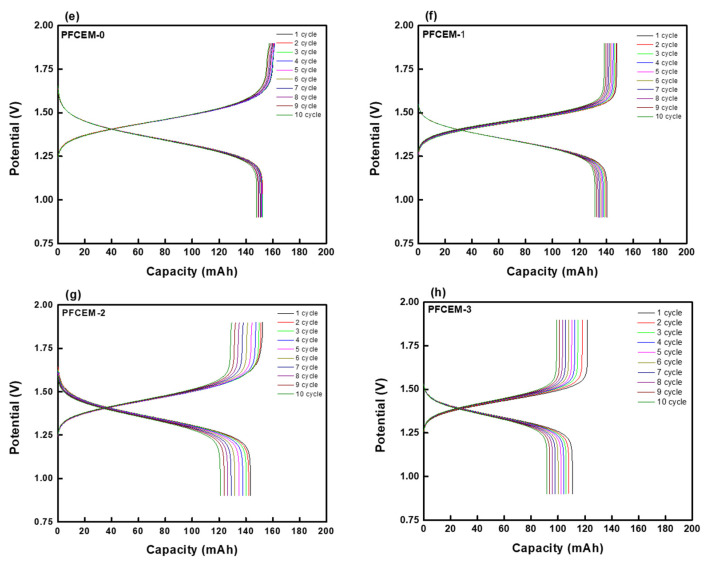
The charge–discharge curves in VRFB systems employing different IEMs ((**a**) PFAEM-0, (**b**) PFAEM-1, (**c**) PFAEM-2, (**d**) PFAEM-3, (**e**) PFCEM-0, (**f**) PFCEM-1, (**g**) PFCEM-2, and (**h**) PFCEM-3, respectively).

The VRFB performance evaluation results with various IEMs are summarized in Figure 11 and Table 6. The results show a tendency to increase the VE by increasing the VBC or Sty molar ratio of the membrane owing to the reduced electrical resistance [52]. As a result, PFAEM-1 and PFCEM-1 having the highest fluorine content exhibited the lowest VE among the membranes tested, but did not show a significant difference from other membranes. On the other hand, the CE was shown to increase by elevating the fluorine content in the membrane, and therefore, PFAEM-1 and PFCEM-1 showed the highest values. The capacity loss of the VRFB showed a tendency to increase as the number of cycles increased, and it is known that this capacity loss originates from the crossover of the active materials through the IEMs and the irreversibility of the oxidation-reduction reactions [53]. In conclusion, PFAEM-1 and PFCEM-1, which have the most fluorine content, showed the highest EE values, and it was confirmed that they had better charge–discharge performance than the commercial membranes. Although it may seem that the difference among the membranes is not large in the results of such a single cell, it is expected that the significant performance difference will occur in a practical system with a large membrane area. Although it is difficult to make an accurate comparison due to the different experimental conditions, the energy efficiency of the fluorine-containing PFIEMs developed in this study was shown to be superior to those of the investigated commercial membranes listed in Table 7.

**Figure 12:**
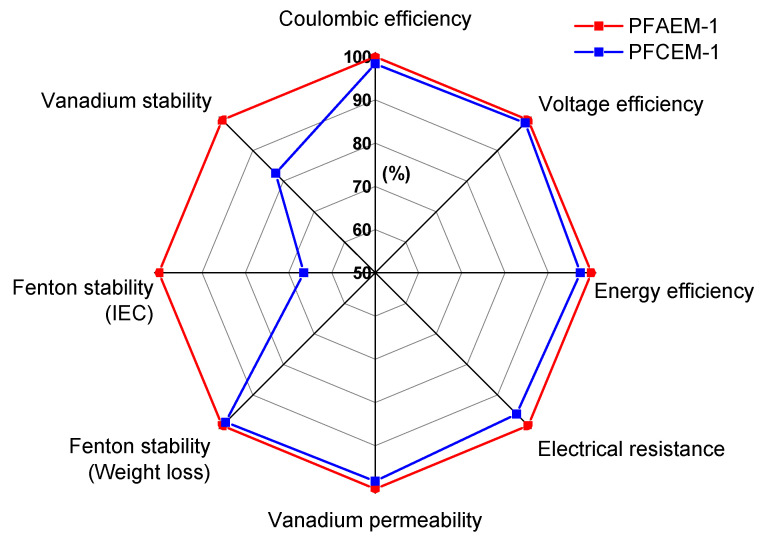
Spider chart for comparing the performances of the PFAEM and PFCEM in a VRFB system.

The characteristics of PFAEM-1 and PFCEM-1, which showed the best VRFB performance, are compared with each other as a spider chart in Figure 12. It can be seen that PFAEM-1 shows better performance than PFCEM-1 in all evaluation criteria. In summary, the PFAEM was not disadvantageous in terms of electrical resistance compared with traditional CEMs when applied to the VRFB due to its thin film thickness. Moreover, it was expected that the vanadium ion crossover could be effectively reduced by the Donnan exclusion, and long-term stability could also be greatly improved by employing the PFAEMs with a fluorine moiety rather than CEMs.

## 4. Conclusions

In this study, novel thin reinforced IEMs were developed by combining a porous PE substrate and an ionomer containing a fluorine moiety for VRFB application. By adjusting the ratio of VBC or Sty and OFPMA, the electrochemical and physicochemical properties of the membranes were effectively controlled. The prepared PFIEMs exhibited superior mechanical properties compared with the commercial membranes despite the thin film thickness owing to the tough physical properties of the porous substrate used. The ion conductivity of the PFIEMs, which contain a large non-ion conducting region owing to the use of the inert porous substrate, was revealed to be lower than that of the commercial membranes. However, the electrical resistance of the PFIEMs could be greatly reduced due to the thin film thickness. Meanwhile, as a result of the evaluation of oxidation stability using Fenton’s reagent, it was confirmed that the oxidation stability of the membranes could be greatly improved through the use of a PE support and the introduction of a fluorine moiety into the filling ionomer. It was also found that the PFIEMs having higher fluorine content exhibit better chemical stability in the vanadium electrolyte, similar to the result of the Fenton oxidation. In addition, the membranes with the highest content of fluorine (i.e., PFAEM-1 and PFCEM-1) showed the lowest vanadium ion permeability, which resulted in the highest current efficiency in the VRFB tests. The PFIEMs also exhibited higher VE compared with the commercial membranes due to the relatively low mass transfer resistance. Overall, PFAEM-1 and PFCEM-1, which have the highest portion of fluorine, showed the highest energy efficiency (89.9% and 87.6%, respectively), and the VRFB performance improvement by using the thin reinforced membranes was expected to increase in a practical system with a large membrane area. Moreover, as a result of comparing the PFAEM and PFCEM, it was concluded that the PFAEM is better than the PFCEM in terms of both charging–discharging performance and durability of VRFB.

## Figures and Tables

**Table 1:** Specifications of the porous PE substrate used for this study [29].

Parameter	Property
Structure	Single layer
Composition	Polyethylene
Thickness (μm)	25
Gurley (s)	21
Porosity (%)	40
*T_m_* (°C)	138

**Table 2:** Composition of monomers for fabricating PFAEMs and PFCEMs.

Membrane	Monomer Mole Ratio	Weight Ratio	
	VBC:Sty:OFPMA	Cross-Linker (DVB)	Initiator (BPO)
PFAEM-0	3:1:0	20 wt%	2 wt%
PFAEM-1	2:0:1		
PFAEM-2	3:0:1		
PFAEM-3	4:0:1		
PFCEM-0	0:1:0	20 wt%	2 wt%
PFCEM-1	0:2:1		
PFCEM-2	0:3:1		
PFCEM-3	0:4:1		

**Table 3:** Ionomer contents in prepared PFIEMs.

**Membrane**	**PFAEM-0**	**PFAEM-1**	**PFAEM-2**	**PFAEM-3**
Ionomer content (wt%)	40.7	41.3	42.6	41.3
**Membrane**	**PFCEM-0**	**PFCEM-1**	**PFCEM-2**	**PFCEM-3**
Ionomer content (wt%)	44.1	44.0	44.1	43.6

**Table 4:** Tensile strength and elongation at break of commercial IEMs, porous substrate, and PFIEMs.

Membrane	Thickness (μm)	Tensile Strength (MPa)	Elongation at Break (%)
AMX (Astom Corp.)	140	29.47	15.60
CMX (Astom Corp.)	160	25.96	18.86
Porous substrate	25	108.5	138.4
PFAEM-1	25	136.6	176.5
PFCEM-1	25	129.9	148.0

**Table 5:** Various properties of commercial membranes and PFIEMs.

Membrane	Thickness (μm)	WU (%)	SR (%)	IEC (meq./g)	*σ* (mS/cm)	MER (Ω·cm^2^)	Transport Number (-)	Contact Angle (°)	$k_{VO^{2+}}$ (× 10^−7^, m/s)
AMX	140	22.4	16.8	1.54	5.24	2.67	0.988	54.14	3.27
PFAEM-0	25	23.8	20.7	2.02	3.97	0.63	0.988	-	2.17
PFAEM-1	25	12.8	19.3	1.34	1.58	1.58	0.978	58.17	1.18
PFAEM-2	25	14.8	20.0	1.67	1.87	1.34	0.985	-	2.57
PFAEM-3	25	16.8	20.9	2.00	2.69	0.93	0.991	-	2.73
CMX	160	27.1	14.8	1.89	5.93	2.70	0.977	45.72	6.54
PFCEM-0	25	17.1	12.7	2.26	4.31	0.58	0.991	-	3.97
PFCEM-1	25	13.1	10.4	1.96	1.52	1.64	0.979	52.53	3.27
PFCEM-2	25	15.7	15.1	2.47	1.82	1.37	0.983	-	4.47
PFCEM-3	25	18.3	17.1	2.74	2.16	1.16	0.987	-	5.39

**Table 6:** Battery efficiencies of commercial membranes and PFIEMs.

Membrane	CE (%)	VE (%)	EE (%)
AMX	92.6	93.2	86.2
PFAEM-0	95.0	94.6	89.9
PFAEM-1	96.4	93.2	89.9
PFAEM-2	94.7	93.4	88.4
PFAEM-3	93.3	94.4	88.0
CMX	90.9	91.3	83.0
PFCEM-0	94.3	92.1	86.8
PFCEM-1	94.8	92.3	87.6
PFCEM-2	93.5	92.5	86.5
PFCEM-3	92.4	93.5	86.3

**Table 7:** VRFB performances of various commercial CEMs and AEMs.

Membrane	Type	Company	CE (%)	VE (%)	EE (%)	Current Density (mA/cm^2^)	Ref.
Nafion 117	CEM	DuPont	85.7	92.5	79.3	30	[54]
Nafion 212	CEM	DuPont	89.6	84.2	75.5	40	[55]
NEPEM115	CEM	Kerun	88.6	85.7	78.5	60	[11]
NR 212	CEM	DuPont	89.2	88.8	79.2	50	[56]
N 115	CEM	DuPont	90.5	85.6	82.8	20	[56]
FAP-PP-475	AEM	Fumatech	92.6	85.0	78.7	60	[11]
FAP-PE-420	AEM	Fumatech	91.0	86.0	78.0	60	[11]
APS	AEM	Asahi Glass	89.3	87.0	77.7	60	[11]

## Data Availability

Not applicable.
